# Supplementary material for: Significance of LIF/LIFR Signaling in the Progression of Obesity-Driven Triple-Negative Breast Cancer
Source: Cancers (Basel). 2024 Oct 28;16(21):3630. doi: 10.3390/cancers16213630 (PMC11545110; doi:10.3390/cancers16213630)
Supplement: Supplementary file 1 [file cancers-16-03630-s001.zip › cancers-3177487-supplementary.pdf]

Supplementary table S1: List of primers used for the study

Gene name	Forward Primer	Reverse Primer
STAT1	ATCAGGCTCAGTCGGGGAATA	TGGTCTCGTGTTCTCTGTTCT
BCL2	GGTGGGGTCATGTGTGTGG	CGGTTCAGGTACTCAGTCATCC
C-MYC	GGCTCCTGGCAAAAGGTCA	CTGCGTAGTTGTGCTGATGT
JUNB	ACGACTCATACACAGCTACGG	GCTCGGTTTCAGGAGTTTGTAGT
TIMP1	CTTCTGCAATTCCGACCTCGT	ACGCTGGTATAAGGTGGTCTG
SOCS2	TTAAAAGAGGCACCAGAAGGAAC	AGTCGATCAGATGAACCACACT
BCL3	AACCTGCCTACACCCCTATAC	CACCACAGCAATATGGAGAGG
HIF1A	CACCACAGGACAGTACAGGAT	CGTGCTGAATAATACCACTCACA
MCL-1	GTAATAACACCAGTACGGACGG	CCACAAACCCATCCTTGGAAG
AKT1	AGCGACGTGGCTATTGTGAAG	GCCATCATTCTTGAGGAGGAAGT
PIM1	GAGAAGGACCGGATTTCCGAC	CAGTCCAGGAGCCTAATGACG
LIFR	TGACTGCATTGCACAGATGA	CCTTTTTCTGGCTATTTACTTGATTC
ID1	CTGCTCTACGACATGAACGG	GAAGGTCCCTGATGTAGTCGAT
ID2	AGTCCCGTGAGGTCCGTTAG	AGTCGTTCATGTTGTATAGCAGG
TNFRSF1A	TCACCGCTTCAGAAAACCACC	GGTCCACTGTGCAAGAAGAGA
18S rRNA	GCTTAATTTGACTCAACACGGGA	AGCTATCAATCTGTCAATCCTGTC
Actin	AAAGACCTGTACGCCAACAC	GTCATACTCCTGCTTGCTGAT
GAPDH	TCGACAGTCAGCCGCATCT	CTAGCCTCCCGGGTTTCTCT

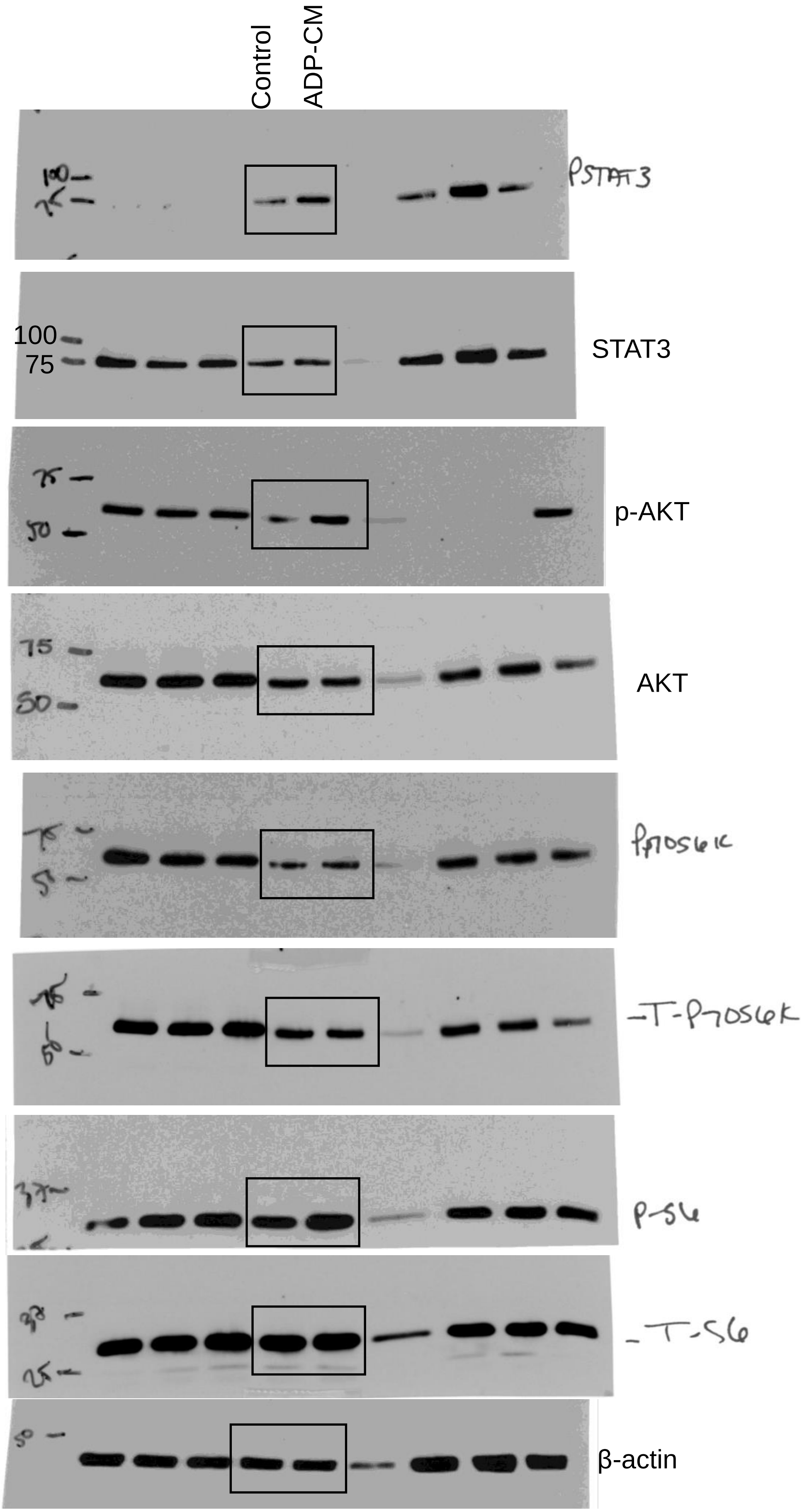

Figure S1. Uncropped Western blots of Figure. 1C

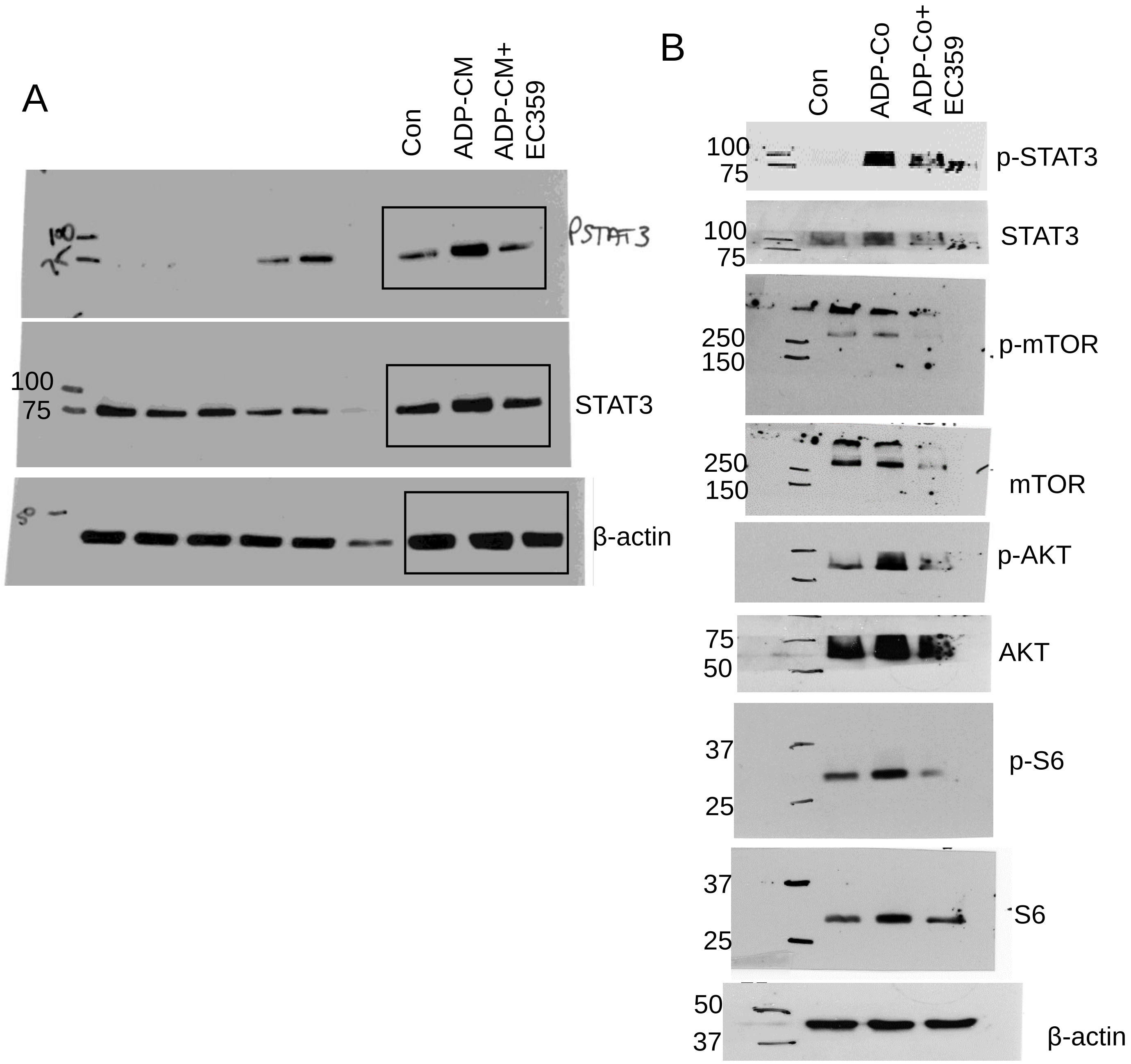

Figure S2. Uncropped Western blots of Figure. 2C (A), and 2D (B)

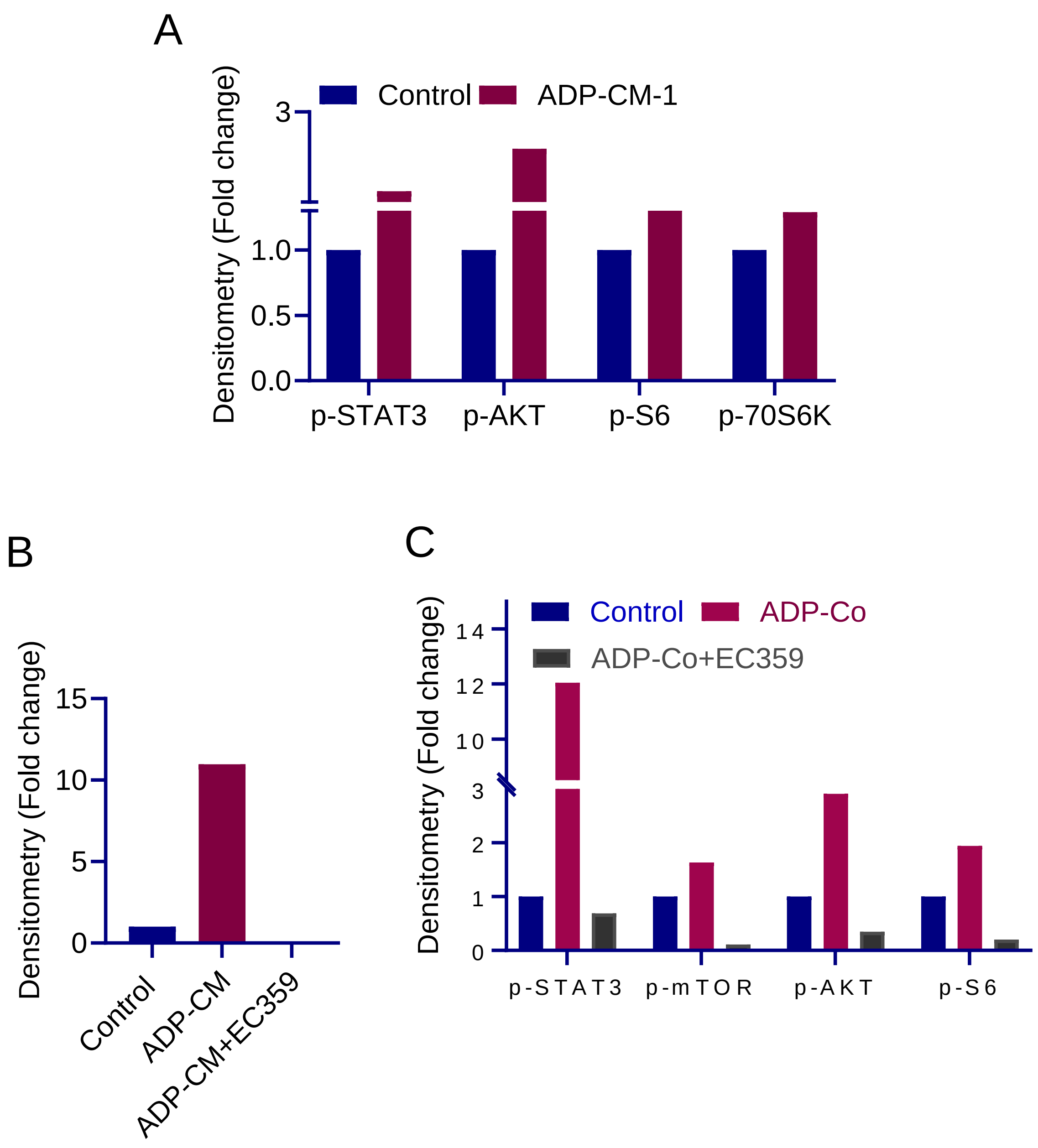

Figure S3. The bar graphs represent the densitometric quantification of Fig. 1C (A), Fig. 2C (B), and Fig. 2D (C) Western blots.

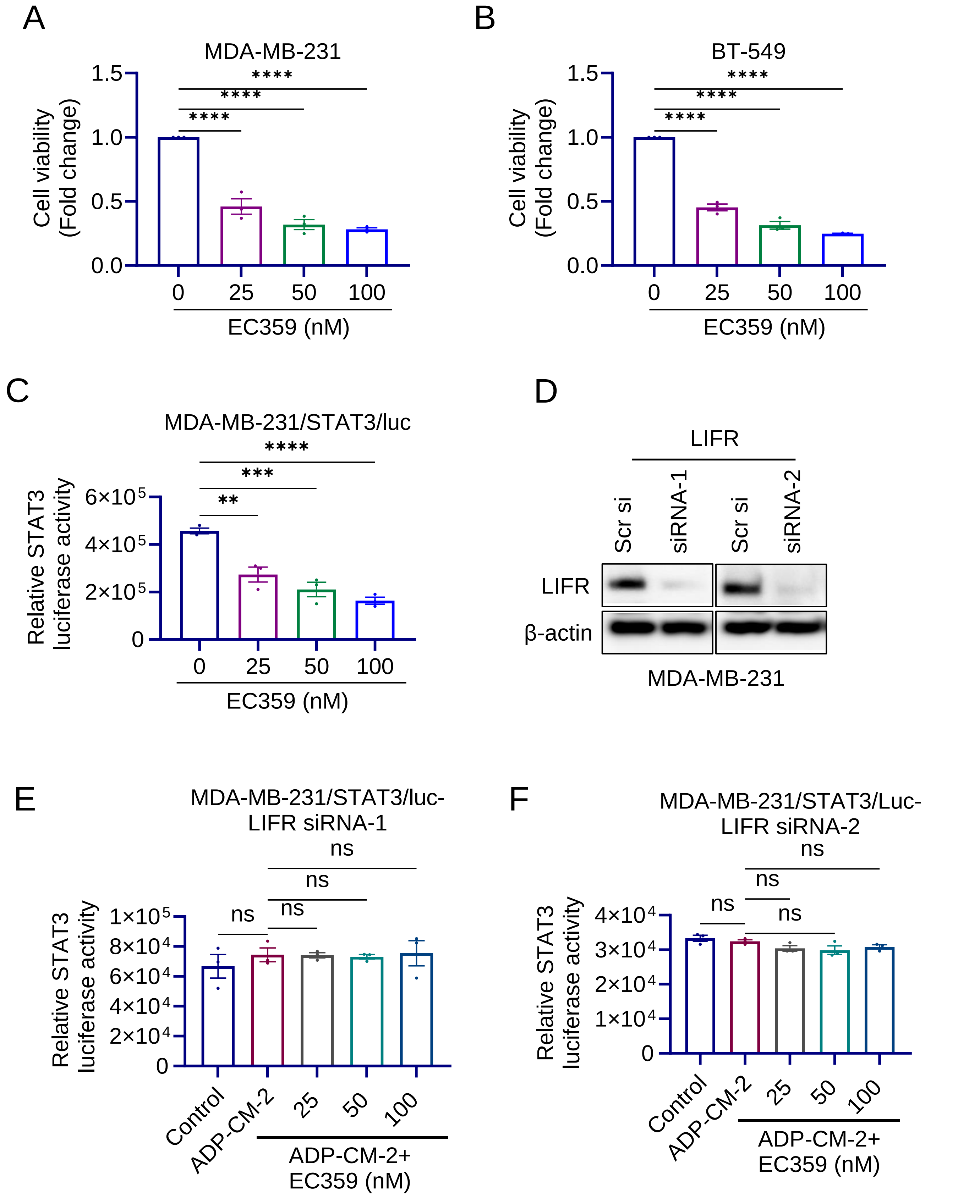

Figure S4. The effects of EC359 treatment on TNBC cell viability (A,B) and STAT3 reporter activity (C) were determined by MTT and luciferase assays. D, The effectiveness of LIFR siRNA in reducing LIFR expression was demonstrated through Western blot analysis. E, F. The effect of ADP-CM on LIFR knockdown cells was evaluated through STAT3 reporter activity assays

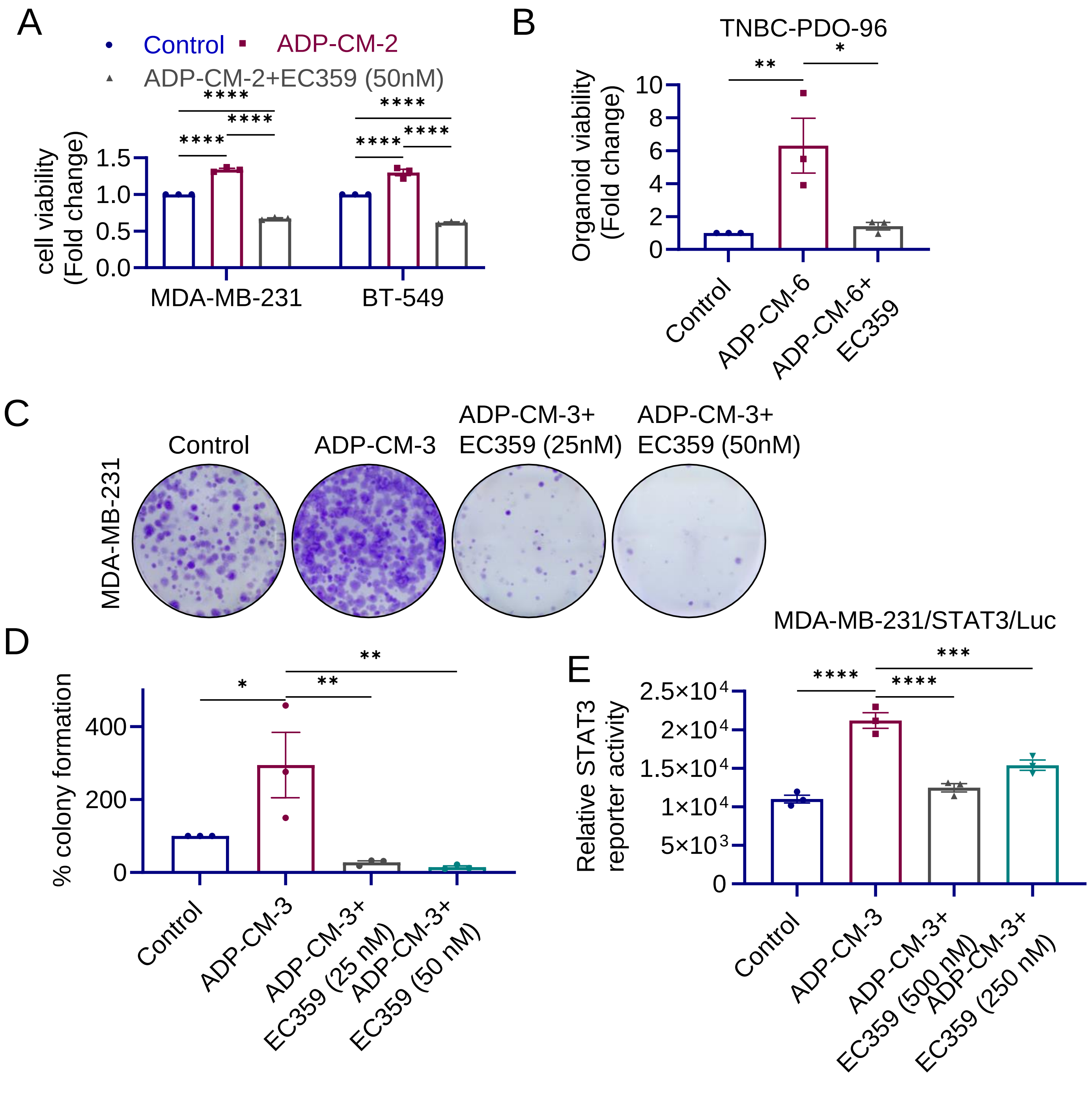

Figure S5. The effects of EC359 treatment against adipose (A) conditions on TNBC cell viability were determined by MTT assays. Organoids generated from TNBC-PDX-96 were cultured in ADP-CM and treated with 1000 nM of EC359. B, Organoids viability of were measured after 7 days of treatment using CellTiter-Glo® 3D Cell Viability Assay. C, The effects of ADP-CM and ADP-CM+EC359 on adiposity-induced cell survival of TNBC cells was measured using colony formation assays. C, Representative images of colonies and its quantification (D) were shown. E, TNBC cells that stably express the STAT3 reporter were incubated with ADP-CM in the presence or absence of EC359 and the reporter activity was measured after 24 hrs. *p<0.05; **p<0.01; ***p<0.001; ****p<0.0001.

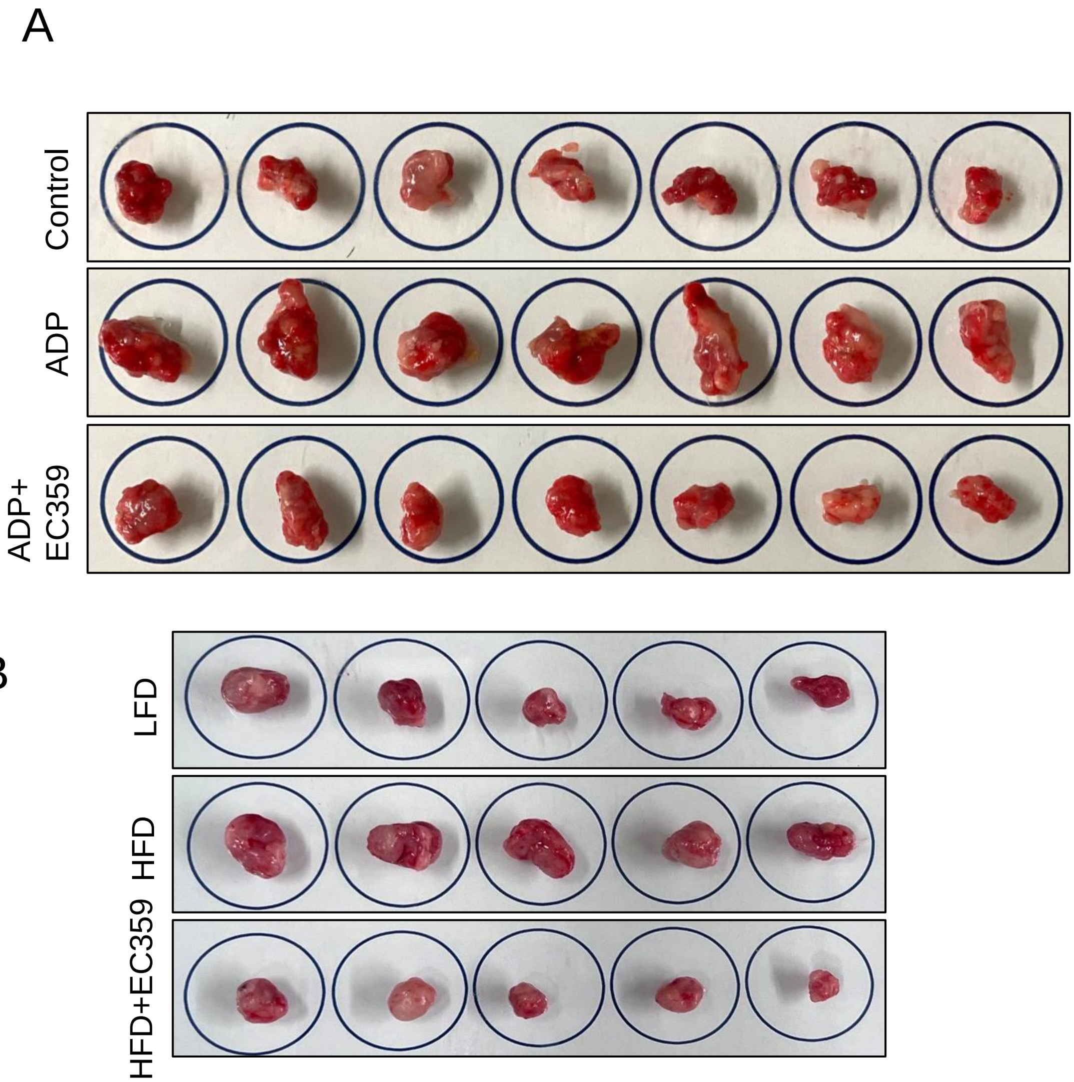

Figure S6: A, MDA-MB-231 xenograft tumor images from control, ADP and ADP+EC359 were shown. B, MDA-MB-231 xenograft tumor images from LFD, HFD and HFD+EC359 were shown.

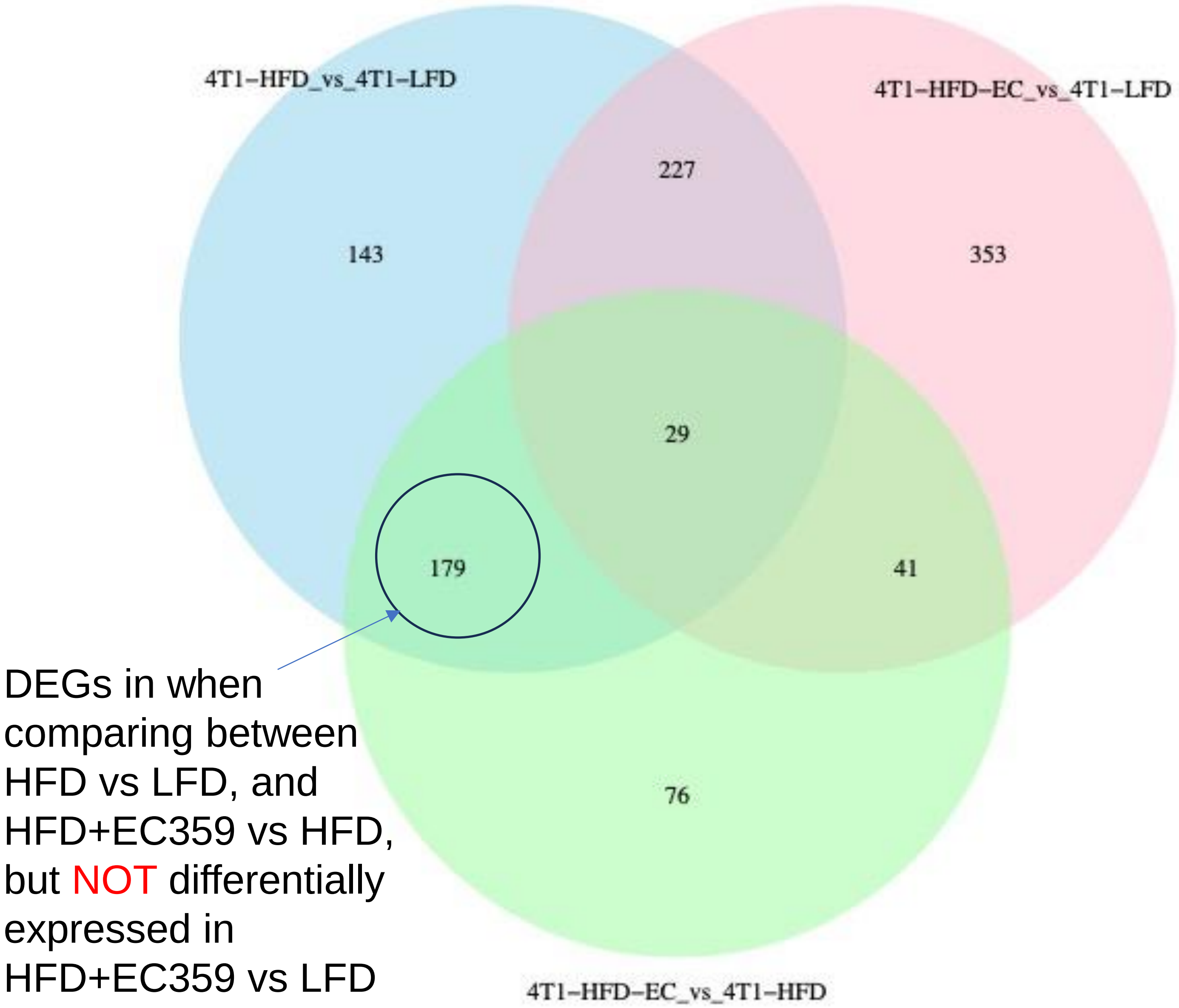

Figure S7. A Venn diagram illustrating the overlapping differentially expressed genes (DEGs) among the 4T1 groups treated with LFD, HFD, and HFD + EC359, based on RNA-seq analysis.

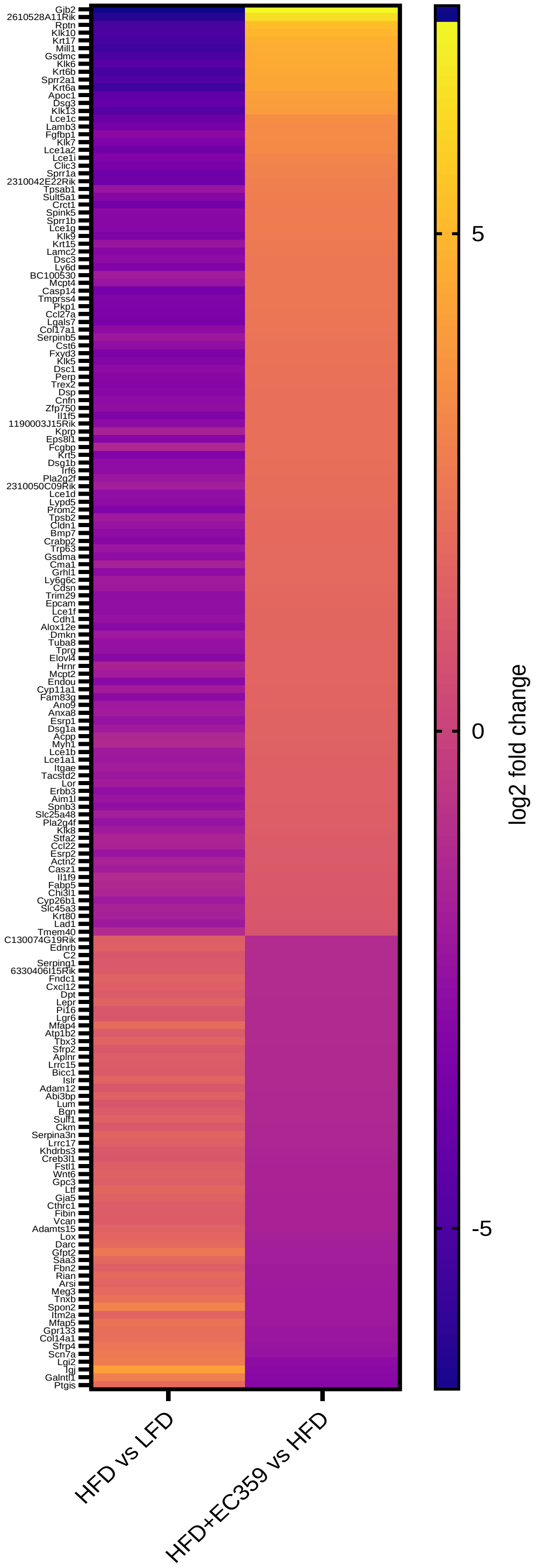

Figure S8. A heatmap displaying the uniquely upregulated and downregulated differentially expressed genes (DEGs) in comparisons between HFD versus LFD and HFD + EC359 versus HFD, based on RNA-seq analysis.
